# The Influence of Industry Affiliation on Randomized Controlled Trials of Platelet-Rich Plasma for Knee Osteoarthritis

**DOI:** 10.1177/03635465221140917

**Published:** 2023-01-03

**Authors:** Canhnghi N. Ta, Rajiv Vasudevan, Brendon C. Mitchell, Robert A. Keller, William T. Kent

**Affiliations:** * Department of Orthopaedic Surgery, University of California, San Diego, San Diego, California, USA; † School of Medicine, University of California, San Diego, San Diego, California, USA; ‡ OrthoCarolina, Charlotte, North Carolina, USA; Investigation performed at the Department of Orthopaedic Surgery, University of California, San Diego, San Diego, California, USA

**Keywords:** platelet-rich plasma, knee osteoarthritis, industry affiliation, industry association, randomized controlled trial

## Abstract

**Background:**

Industry funding and corporate sponsorship have played a significant role in the advancement of orthopaedic research and technology. However, this relationship raises concerns for how industry association may bias research findings and influence clinical practice.

**Purpose:**

To determine whether industry affiliation plays a role in the outcomes of randomized controlled trials (RCTs) investigating platelet-rich plasma (PRP).

**Study Design:**

Meta-analysis; Level of evidence, 2.

**Methods:**

A search of the PubMed, Cochrane, and MEDLINE databases for RCTs published between 2011 and the present comparing PRP versus hyaluronic acid, corticosteroid, or placebo for the treatment of knee osteoarthritis was performed. To determine industry affiliation, the conflict of interest, funding, and disclosure sections of publications were assessed, and all authors were assessed through the American Academy of Orthopaedic Surgeons disclosure database and the Centers for Medicare & Medicaid Services open payments database. Studies were classified as industry affiliated (IA) or non–industry affiliated (NIA). The outcomes of each study were rated as favorable, analogous, or unfavorable according to predefined criteria.

**Results:**

A total of 37 studies (6 IA and 31 NIA) were available for analysis. Overall, 19 studies (51.4%) reported PRP as favorable compared with other treatment options, while 18 studies (48.6%) showed no significant differences between PRP and other treatment methods. There was no significant difference in qualitative conclusions between the IA and NIA groups, with the IA group having 3 favorable studies and 3 analogous studies and the NIA group having 16 favorable studies and 15 analogous studies (*P* = .8881). When comparing IA versus NIA studies using 6- and 12-month Western Ontario and McMaster Universities Arthritis Index and International Knee Documentation Committee scores, there were no significant differences in outcomes.

**Conclusion:**

The results of this study demonstrated that qualitative conclusions and outcome scores were found to not be associated with industry affiliation. Although the results of this study suggest that there is no influence of industry involvement on RCTs examining PRP, it is still necessary to carefully evaluate pertinent commercial affiliations when reviewing recommendations from studies before adopting new treatment approaches, such as the use of PRP for knee osteoarthritis.

Orthopaedic surgery is a rapidly evolving field that has benefited from profound innovations in biomedical research and technology. Industry funding and sponsorship have played an integral role in this advancement. However, concerns remain that industry funding may bias research findings and eventually influence evidence-based practice. In 2012, a total of $116.5 billion was spent on biomedical research, with industry funding representing the majority of the contribution (58%).^41^ Conversely, government sponsorship has seen a progressive decline, as demonstrated by the steady decrease in National Institutes of Health funding since 2004.^41^ Furthermore, the percentage of orthopaedic researchers reporting conflicts of interest in the scientific literature markedly increased from 3% to 51.9% between 1984 and 2021.^10,40,62^ To ensure that evidence-based medicine remains unbiased, critical analysis of industry-funded studies is warranted.

Novel intra-articular injection therapies, particularly biologics, are an emerging treatment method for knee osteoarthritis.^26^ In 2010, osteoarthritis was the second most common medical condition, affecting 10% to 13% of adults aged ≥60 years in the United States, and accounted for the highest medical expenditure at $16.5 billion.^62^ Although the exact mechanism of platelet-rich plasma (PRP) remains unknown, PRP has been shown to stimulate chondrocyte proliferation and the biosynthesis of collagen and proteoglycans.^2^

Several studies have investigated the influence of industry involvement on study outcomes. One review of all randomized controlled trials (RCTs) published in 5 major orthopaedic journals between 2002 and 2004 found that industry-affiliated (IA) studies were 10.9 times more likely to report positive findings compared with non–industry-affiliated (NIA) studies.^31^ More recent studies have noted similar patterns in major journals across multiple disciplines of orthopaedic research.^17,34,40,52^ A 2013 review of all RCTs up to 2010 investigating hyaluronic acid (HA) versus placebo found that studies with industry affiliation reported favorable or neutral outcomes, with no studies reporting negative outcomes being present.^47^ Interestingly, studies without industry affiliation had a much higher rate of negative outcomes of 35%.^47^

To our knowledge, there are no studies that have characterized the influence of industry involvement on the outcomes of trials assessing the efficacy of intra-articular PRP for the treatment of knee osteoarthritis. The goal of our study was to determine whether industry affiliation plays a role in the outcomes of RCTs investigating PRP versus other intra-articular injectables, such as HA, corticosteroid, and placebo.

## Methods

**Figure 1.**
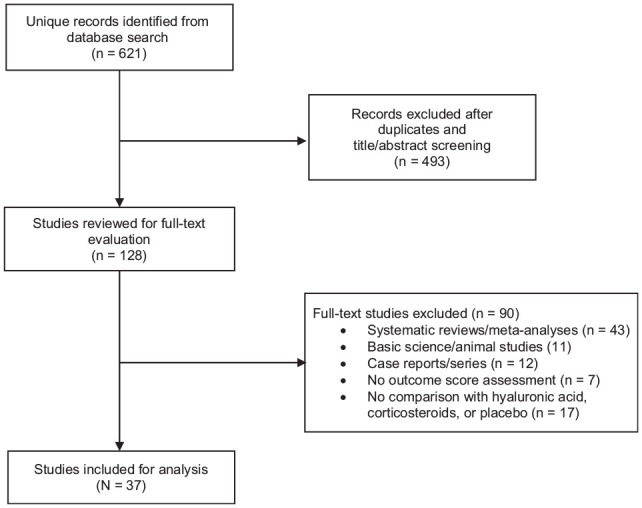
Flowchart of inclusion and exclusion criteria for study selection.

A search of the PubMed, Cochrane, and MEDLINE databases for “platelet-rich plasma” and “knee osteoarthritis” was performed by 2 independent reviewers (C.N.T. and R.V.) on December 1, 2021. The search included RCTs of level 1 or 2 evidence published between 2011 and the present comparing PRP versus HA, corticosteroid, or placebo for the treatment of knee osteoarthritis. Clinical outcomes included patient-reported outcome measure (PROM) scores, such as the Western Ontario and McMaster Universities Arthritis Index (WOMAC) and International Knee Documentation Committee (IKDC) scores. Exclusion criteria included basic science or animal studies (n = 11); systematic reviews or meta-analyses (n = 43); case reports or case series (n = 12); studies with no outcome assessments (n = 8); studies with no comparison with either HA, corticosteroid, or placebo (n = 17); and non–level 1 or 2 studies (n = 493) (Figure 1).

### Determination of Industry Affiliation

To determine industry affiliation, we assessed the conflict of interest section of each study. All authors were also evaluated using the American Academy of Orthopaedic Surgeons (AAOS) disclosure database (http://disclosure.aaos.org) and the Centers for Medicare & Medicaid Services (CMS) open payments database (http://openpaymentsdata.cms.gov).^3,43^ Studies were classified as IA based on the presence of financial conflicts of interest, such as self-reported conflicts that were directly related to a manufacturer of the treatment method (ie, PRP system manufactured by a specific company), direct industry funding of the study, and/or relevant disclosures found in the online AAOS disclosure database or CMS open payments database from the year of publication or before. Studies were otherwise classified as NIA. For studies without authors from the United States, the CMS open payments database could not be utilized, and therefore, industry affiliation could only be determined via reports in the AAOS disclosure database.

Financial conflicts of interest by industry association were identified as licensing or royalty fees, consultant fees, paid advisory positions, speaker fees, employees, stock options, or research funding from companies that synthesize PRP or manufacture devices to administer PRP. This comprehensive method of determining affiliation was utilized because of the impracticality of guaranteeing a direct relationship between a conflict of interest and PRP. However, similar methods have been described in previously published studies.^21,34^ If any of the authors were found to have relevant conflicts of interest, the study was considered to be IA. Associations with and funding from other nonindustry entities, such as government agencies or academic institutions, were not categorized as financial conflicts of interest.

### Determination of Outcome

The outcomes of each study were rated as favorable, analogous, or unfavorable according to predefined criteria based on previously published protocols.^21,40^ Studies with favorable outcomes were those that showed the superior results of PRP over HA, corticosteroid, or placebo. Studies with unfavorable outcomes were those in which PRP resulted in inferior outcomes compared with the other treatment options. Studies with analogous results were those that reported no significant clinical differences between PRP and other treatment options. Each study was assessed by 2 reviewers, who were blinded to study funding and each other’s rating (C.N.T. and R.V.). After individual determinations, reviewers deliberated on the outcome ratings until a consensus was reached for each study.

An additional subanalysis was performed to compare studies by specific outcome scores. In a preliminary analysis, the most common outcome scores that were measured and compared were IKDC and WOMAC scores at 6 and 12 months. The outcomes were again rated as favorable, analogous, or unfavorable but were based on statistical significance when comparing scores using PRP versus HA, corticosteroid, or placebo (*P* < .05 or *P* > .05).

### Statistical Analysis

Statistical analysis was performed using R statistical software (Version 4.1.2; R Foundation for Statistical Computing). Descriptive statistics were reported as percentages. Ratings, PROM scores, and levels of evidence were compared between IA and NIA studies using chi-square test. *P* < .05 was considered statistically significant.

## Results

The initial search strategy resulted in 621 studies. After removing duplicates and studies by title and abstract screening, 128 studies were analyzed for a full-text evaluation. After a review by the same 2 independent raters, a total of 37 studies met inclusion criteria and were included for analysis (Figure 1).

There were 6 (16.2%) IA and 31 (83.8%) NIA studies. Overall, 11 of the 31 NIA studies (35.5%) reported conflicts of interest, but these were determined to be nonindustry associations, such as grants from a ministry of health or support from academic research centers.^‖^ One study was found to have a potential industry association via the AAOS disclosure database, which was not disclosed in the published study.^32^

In total, 19 studies (51.4%) reported PRP as favorable compared with other treatment approaches, while 18 studies (48.6%) showed no significant differences between PRP and other treatment options. No studies found poorer outcomes with PRP compared with HA, corticosteroid, or placebo. All studies were of level 1 (70.3%) or level 2 (29.7) evidence, with no statistically significant difference between IA and NIA studies (*P* = .4443).

When comparing general outcomes by evaluating the discussion and conclusion sections of each publication, we found the IA group had 3 favorable studies and 3 analogous studies while the NIA group had 16 favorable studies and 15 analogous studies (*P* = .8881) (Table 1). In the subanalysis comparing IA versus NIA studies using PROMs, 26 of the 37 included studies evaluated WOMAC and IKDC scores at 6 and 12 months. Among these studies, there were no significant differences between outcome scores at either time point (Table 2).

**Table 1:** Comparison of Qualitative Conclusions

Outcome	Affiliated (n = 6), n	Nonaffiliated (n = 31), n	*P* Value
Favorable	3	16	.8881
Analogous	3	15	

**Table 2:** Comparison of Outcome Scores^*a*^

Outcome	Affiliated, n	Nonaffiliated, n	*P* Value
6-mo WOMAC (n = 18)			.8849
Favorable	1	13	
Analogous	1	3	
12-mo WOMAC (n = 11)			.3869
Favorable	1	9	
Analogous	1	0	
6-mo IKDC (n = 10)			.6726
Favorable	2	5	
Analogous	2	1	
12-mo IKDC (n = 7)			.7409
Favorable	1	3	
Analogous	2	1	

a IKDC, International Knee Documentation Committee; WOMAC, Western Ontario and McMaster Universities Arthritis Index.

The majority of included studies were conducted outside of the United States (91.9%). All 3 US-based studies were found to have a potential corporate affiliation compared with 3 (8.1%) of the non-US studies.^8,9,18,32,53,59^ A complete summary of included studies, including the details of industry affiliation, can be found in Table 3.

**Table 3:** Summary of Included Studies^*a*^

First Author (Year)	Level of Evidence	Affiliated	Conflicts of Interest/ Industry Affiliations	Outcome Rating
Cole^8^ (2017)	1	Yes	Research support from Aesculap/B. Braun, Arthrex, Athletico, Cytori, Medipost, National Institutes of Health, Ossur, Smith & Nephew, Tornier, and Zimmer; intellectual property royalties from and paid consultant for Arthrex, DJ Orthopedics, Regentis, Zimmer, Smith & Nephew, and Tornier; stock or stock options in Carticept; research support/material support from and paid presenter or speaker for Arthrex and Kensey Nash	Favorable
Di Martino^9^ (2019)	1	Yes	Stock or stock options in CartiHeal; speaking and consulting fees from Fidia, Finceramica, Zimmer Biomet, CartiHeal, and GreenBone; institutional support from CartiHeal, DSM Biomedical, Fidia, Finceramica, GreenBone, IGEA Clinical Biophysics, Piramal/Smith & Nephew, and Zimmer Biomet	Analogous
Filardo^18^ (2015)	1	Yes	Institutional support from and consultant for Fidia and Zimmer Biomet	Analogous
Smith^53^ (2016)	1	Yes	Consultant for Arthrex (study supported by Arthrex)	Analogous
Vaquerizo^59^ (2013)	1	Yes	Active contribution from BTI Biotechnology Institute	Favorable
Kon^32^ (2011)	2	Yes	Paid consultant for BIOVIIIx, CartiHeal, Fidia, Geistlich, GreenBone, Mastelli, and Zimmer Biomet; research support from CartiHeal	Favorable
Ahmad^1^ (2018)	1	No	No financial conflicts of interest	Favorable
Bansal^4^ (2021)	1	No	No financial conflicts of interest	Favorable
Buendía-López^6^ (2018)	1	No	No financial conflicts of interest	Favorable
Cerza^7^ (2012)	1	No	No financial conflicts of interest	Favorable
Dório^11^ (2021)	2	No	No financial conflicts of interest	Analogous
Duymus^13^ (2017)	1	No	No financial conflicts of interest	Favorable
Elik^14^ (2020)	1	No	No financial conflicts of interest	Analogous
Elksniņš-Finogejevs^15^ (2020)	1	No	No financial conflicts of interest	Analogous
Filardo^19^ (2012)	1	No	Supported by Ricerca Finalizzata (2009)	Analogous
Forogh^20^ (2016)	1	No	No financial conflicts of interest	Favorable
Görmeli^22^ (2017)	1	No	No financial conflicts of interest	Analogous
Huang^25^ (2019)	1	No	No financial conflicts of interest	Analogous
Joshi Jubert^28^ (2017)	2	No	Financial support for study (grant) from Ministry of Health, Social Policy and Equality	Analogous
Kesiktas^29^ (2020)	1	No	No financial conflicts of interest	Analogous
Lana^33^ (2016)	1	No	No financial conflicts of interest	Favorable
Lin^36^ (2019)	1	No	Funded by Kaohsiung Veterans General Hospital (research grant VGHKS 103-075) and registered with Government Research Bulletin	Favorable
Louis^39^ (2018)	2	No	Free Durolane	Analogous
Montañez-Heredia^41^ (2016)	2	No	No financial conflicts of interest	Analogous
Nabi^42^ (2018)	1	No	Supported by Anesthesiology Research Center at Guilan University of Medical Sciences	Favorable
Patel^44^ (2013)	1	No	Financial support from Prof. D.S. Grewal Memorial Orthopaedics Society and Indian Arthroplasty Association; PRP prepared and provided by Department of Transfusion Medicine at Postgraduate Institute of Medical Education and Research	Favorable
Paterson^45^ (2016)	2	No	Royalties from sales of educational osteoarthritis DVD and sales of osteoarthritis shoe (neither related to work in study)	Analogous
Pishgahi^46^ (2020)	2	No	Financial support from Physical Medicine and Rehabilitation Research Center at Tabriz University of Medical Sciences	Analogous
Raeissadat^49^ (2015)	1	No	No financial conflicts of interest	Favorable
Raeissadat^48^ (2021)	1	No	Funded by Vice Chancellor for Research of Shahid Beheshti University of Medical Sciences (no role in design of study; collection, analysis, and interpretation of data; and writing of article)	Favorable
Say^51^ (2013)	1	No	No financial conflicts of interest	Favorable
Spaková^54^ (2012)	2	No	No financial conflicts of interest	Favorable
Su^55^ (2018)	2	No	No financial conflicts of interest	Analogous
Tavassoli^56^ (2019)	1	No	Supported by Vice Chancellor for Research and Technology of Babol University of Medical Sciences (No. 970568); PRP prepared with Rooyagen Kit (Arya Mabna Tashkhis)	Favorable
Uslu Güvendi^58^ (2017)	2	No	No financial conflicts of interest	Analogous
Wu^60^ (2018)	1	No	Supported by Ministry of Science and Technology (grant No. MOST 104-2314-B-016-050)	Favorable
Yu^61^ (2018)	2	No	No financial conflicts of interest	Analogous

a PRP, platelet-rich plasma.

## Discussion

The findings from this study demonstrate the absence of an association between qualitative conclusions or PROM scores and industry affiliation in level 1 and 2 studies assessing the efficacy of intra-articular PRP for the treatment of knee osteoarthritis. We also demonstrate that the majority of RCTs reported positive or noninferior outcomes with PRP compared with other injectables.

There has been a significant rise in conflict of interest reporting, particularly in the orthopaedic literature. Previous studies have established an association between commercial support and a higher likelihood of reporting favorable outcomes with various treatment methods.^17,31,34,40,47,52^ For example, in a retrospective review of spine publications, Shah et al^52^ found that the odds ratio of industry-funded studies reporting positive results was 3.3 times that of studies with other funding sources. Printz et al,^47^ in a review examining the relationship between financial conflicts of interest and outcomes using HA for knee osteoarthritis, found that the majority of publications were industry funded (63%) and that qualitative conclusions were significantly associated with financial conflicts of interest. The results of the current study suggest that there is no association between industry funding and qualitative conclusions in RCTs evaluating PRP, as outcome ratings were distributed as roughly 50% favorable and 50% analogous for both the IA and NIA groups. Many of the previous industry-sponsored studies have used categorical outcome scores; however, we assessed outcomes using continuous variables and showed no difference in the distribution of PROM scores between the 2 groups.^17,21,47^

A recent review by Mayo et al^40^ reported similar findings to those of our study in which the authors found no statistical association between the frequency of favorable outcomes using autologous chondrocyte implantation and the presence of financial conflicts of interest, country of authorship, or level of evidence. A strength of their study was the use of both the AAOS disclosure database and the CMS open payments database to better determine the financial conflicts of interest. However, a large majority of their included studies were of level 3 or 4 evidence and were published outside of the United States. We encountered similar limitations. However, a significant advantage of the current study was the inclusion of publications of only level 1 or 2 evidence, specifically RCTs. The lack of an association between industry affiliation and study outcomes noted in the current study may reflect the design of the included studies. We suspect that non-RCT study designs, which are inherently more susceptible to bias, may be at a greater risk of industry influence. To the best of our knowledge, there are no studies that have directly compared the effect of industry affiliation on outcomes in RCTs versus non-RCTs. This may be of interest for future studies. However, our findings are reflective of previous literature in which industry collaboration was shown to have no association with outcomes in RCTs.^30,37^ In contrast, investigations that included primarily non-RCT studies have demonstrated that industry affiliation is more likely to be associated with positive findings.^17,38^ This highlights the importance of critically evaluating the study design and methodology when looking for bias and interpreting results.

It is notable that industry affiliation is associated with a very low rate of studies with unfavorable or negative outcomes in the orthopaedic literature.^17,34,47,52^ In our review, none of the included studies found PRP to be inferior to HA, corticosteroid, or placebo. In a 2005 review of the spine literature, Shah et al^52^ reported negative results in 7.1% of industry-funded studies. These findings may be attributed to several factors. Publication bias may have played a role in the low rate of unfavorable studies, as it has been demonstrated in the literature that studies with nonsignificant results are significantly less likely to be published.^23,24,27^ Additionally, some studies have reported that sponsored studies are often terminated before completion or publication because of unfavorable results that may have opposed the interests of the industry sponsor.^16,35^ However, it is difficult to determine whether this occurred with studies involving PRP. Moreover, it is plausible that unknown affiliations may exist with the studies in the NIA group. It has been established that discrepancies and underreporting with conflicts of interest remain an issue.^5,12,50^ In a study evaluating financial disclosure accuracy in major surgical trauma journals, Tisherman et al^57^ found that only 16.6% of authors accurately disclosed their financial relationships with industry entities. The present study found that only 1 (2.7%) of the included studies had identifiable financial conflicts of interest, revealed by the AAOS database, that were not included in the self-reported disclosure portion of the publication. The true rate of affiliated studies may be higher. A total of 17 (45.9%) included studies self-reported ≥1 conflicts of interest in the published disclosure section. Only 6 of these studies were found to have a potential industry affiliation with PRP, while the other studies had nonindustry conflicts of interest. Industry sponsorship can manifest in various forms, such as research grants, consultancies, or educational funds, and it was necessary to include all possible sources of corporate affiliation because of any author investment or relationship with a company potentially influencing outcomes. It was also imperative to scrutinize each of the study conflicts for nonindustry associations, such as support from research grants or academic centers. Helpful disclosures were provided as well, with statements indicating that funding bodies played no role in the design of the study; in the collection, analysis, and interpretation of data; and in writing of the article.^48^ Furthermore, a few of the included studies had authors who showed industry-related conflicts of interest in the AAOS database that were not disclosed in the publication. However, the dates of these disclosures in the AAOS database were subsequent to the dates of publication and therefore could not be applied to these studies. Thus, these publications were not included in the IA group.

Another finding of this review was that the vast majority of included studies were published outside of the United States and were found to have no corporate association, while 50.0% of the IA studies were US based. The review from Mayo et al^40^ found similar results in which there was a significantly greater presence of financial conflicts of interest in US studies. This may be attributed to mandated reporting processes in the United States such as the CMS database and the lack thereof in other countries, resulting in higher rates of reporting.

There were multiple limitations to this study. There was inherent subjectivity to the outcome ratings of the studies. To mitigate this, each reviewer was blinded to the affiliation status and the other reviewer’s ratings. The subanalysis conducted to compare outcome scores by statistical significance also provided an objective evaluation and supplemented the primary results. Furthermore, the number of US-based publications in this study was small, and studies with larger sample sizes comparing US versus non-US conflicts of interest are necessary. Another limitation was the relatively small number of IA studies compared with NIA studies. This may be because of the thorough evaluation and scrutiny of each study for valid and pertinent financial conflicts of interest. Multiple studies reported conflicts of interest but were found to be associated with government agencies or academic institutions and thus deemed to be nonindustry associations. A significant drawback was the lack of certain details and capabilities with the online mandated reporting processes. For the AAOS database, only the most recent yearly disclosures are provided, and therefore, conflicts of interest before the publication could not be consistently obtained. In several studies, possible unreported disclosures were found, but they were stated past the publication date and thus could not be determined to be a potential financial conflict of interest. More details and requirements are needed in both the AAOS disclosure database and the self-reported disclosure portions of publications to properly discern how relevant a conflict of interest may be. Additionally, the lack of CMS database reporting for authors outside of the United States was another constraint, with a large majority of the studies being non-US publications. Further regulations on mandated reporting of financial conflicts of interest should be implemented on an international level to increase transparency for both patients and clinicians when evaluating these studies.

In conclusion, the results of this study demonstrated largely favorable and analogous results with PRP compared with other intra-articular injection therapies for knee osteoarthritis in RCTs. Our findings suggest that qualitative conclusions and outcome scores were found to not be associated with industry affiliation. Although the results of this study suggest that there is no influence of industry involvement on RCTs examining PRP, it is still necessary to carefully evaluate pertinent commercial affiliations when reviewing recommendations from studies before adopting new treatment approaches, such as the use of PRP for knee osteoarthritis.

## Footnotes

‖ References 18, 28, 36, 39, 42, 44-46, 48, 56, 60.
